# Special issue of the journal *international economics and economic policy: international economics, climate policy innovations and economic policy (IEEP, issue 2)*

**DOI:** 10.1007/s10368-022-00540-x

**Published:** 2022-06-22

**Authors:** Lucas Bretschger, Paul J. J. Welfens

**Affiliations:** 1 ETH, Institute of Economic Research, Zurich, Switzerland; 2 European Institute for International Economic Relations, University of Wuppertal, Gaußstr. 20, 42119 Wuppertal, Germany

## Introduction

On September 24th, 2021, the European Institute for International Economic Relations (EIIW/University of Wuppertal) hosted an international workshop in the context of marking the Institute’s 25th Anniversary; it had initially been hoped that postponing the workshop from the originally intended date in 2020 to 2021 would allow to have a normal *in person* conference, but COVID-19 dynamics continued to disrupt plans, and the workshop took place belatedly in an online format. Founded at the University of Potsdam, the Institute subsequently moved to the University of Wuppertal where, over many years, the EIIW team and guest researchers have contributed greatly to international economic analysis. This has included work for various International Organizations (e.g., the United Nations, International Monetary Fund) as well as research for governments (and public agencies) in the European Union — including the European Parliament and the European Commission — companies and non-governmental organizations (NGOs) across Europe. The Special Issue presented here provides an exemplary selection of the research in the Institute’s area of expertise. It places an analytical focus on International Economics, climate-policy-related research, International Organizations and economic policy analysis, including some of the first papers with analytical economic findings on the effects of the Russo-Ukrainian war.

This special issue thus reflects key fields of International Economics and climate policy as well as research on key aspects of the war in the Ukraine. In the paper of Werner Roeger and Paul Welfens, there is a clear focus on the macroeconomic effects of import tariffs in a model with multinational firms and foreign direct investment (FDI): A major analytical innovation here is that foreign direct investment is included in a DSGE model alongside trade, while traditional models have emphasized just trade therefore omitting an important channel of modern globalization in OECD countries and Newly Industrialized Countries. Thus, one gets new insights into the effects of import tariffs and protectionism, respectively: for the home country, the foreign country and the world economy. The US-China trade conflict as well as US-EU trade conflicts under the Trump Administration is a potentially relevant background and field of application for the new modelling approach presented.

The contribution of Samir Kadiric analyzes recent developments in the British and European government bond markets with reference to BREXIT. Firstly, the author examines whether the BREXIT referendum affected the risk premium and, secondly, whether there are any changes in terms of risk pricing following the British referendum. There is a significant impact of the BREXIT referendum on the risk premium in various economies. Moreover, regression results suggest that there was a considerable change in risk pricing following the announcement of the referendum result. Credit default risk and risk aversion play a much more important role in the post-referendum period than was the case prior to the vote.

Friedrich Schneider, who has been a pioneer in the international analysis of the dynamics of the shadow economy, presents new results on global changes in the shadow economy: Considering the development of the shadow economy of 36 European and OECD countries over the period 2003–2022 and the effect of the COVID-19 pandemic from 2020 onwards, the average size of the shadow economy of 36 European and OECD countries decreased slightly (relative to gross domestic product). Due to a continued — forecasted — economic recovery in 2022, the average shadow economy of these 36 countries will slightly increase to about 16% of aggregate income — this is the average of all 36 countries included in the analysis. There are considerable differences in the size of the shadow economy across countries; as regards policy monitoring by International Organizations, corruption is often covered in standard approaches; however, shadow economic activities have thus far been given relatively little consideration, despite the fact that in many countries of the world economy, the size of the shadow economy is crucial in economic terms.

Kirill Borissov, Lucas Bretschger, and Aleksei Minabutdinov focus on the topic of capital taxation in a greening economy. By adopting a model of endogenous growth with polluting capital and a fixed budget for aggregate emissions, the authors identify a novel and interesting capital tax paradox. The basic perspective relates to pollution abatement efficiency which is growing over time due to technical progress. The authors take a look at long-run capital and consumption which turn out to be inversely related to the initial stock of capital. As a consequence, capital taxation does not harm the economy but actually raises long run consumption and production: This is the “green capital tax paradox.” The analysis explains this, at first sight, surprising result by the fact that early economic activity is harmful because of high environmental pollution. The contribution also presents novel results on the impact of pollution intensity and the rate of technical progress on the greening of the economy and pollution allowance prices. As regards the quantitative contribution, the authors calibrate the model and study economic growth under different assumptions with respect to basic parameter values. With climate challenge remaining a major policy topic in the world economy, this contribution is highly relevant — and also plays a crucial role at a United Nations level (despite the recent Russo-Ukrainian war possibly changing the agenda in major OECD and G20 countries, respectively, for a transitory period).

The study by Vasily Astrov, Mahdi Ghodsi, Richard Grieveson, Mario Holzner, Michael Landesmann, Olga Pindyuk, Robert Stehrer, and Maryna Tverdostup from the Vienna Institute for International Economic Studies (wiiw) raises crucial questions with respect to the Russio-Ukrainian war: What are the economic effects of the war for Ukraine, Russia, the EU, and the rest of Europe, respectively? The analysis of the authors sheds an interesting light on the immediate consequences on the one hand, but also on the medium-term structural changes caused by this war on the other. The Russian invasion of Ukraine has caused a humanitarian crisis and one should anticipate a major refugee wave in Europe. As Ukraine’s Black Sea ports come under Russian assault, Ukraine has lost its ability to sell more than half of its regular exports. Obviously, Western financial support (possibly including considerable IMF funding as well as EBRD support) will become ever more important as the Russio-Ukrainian war continues. Turning to Russia, sanctions will have a very serious impact on the economy, including the financial sector. Discretionary intervention by the Russian Central Bank and the government, including capital controls, have been employed by Moscow to cope with key international challenges. As a result of the war and the Western sanctions, the rest of Europe faces a rise in already high inflation rates. Energy imports from Russia are crucial for many European countries. Aside from energy, the economic fallout via trade for the rest of Europe is likely to be rather modest. There are several key areas of structural change and lasting impacts for the EU as a result of Russia’s invasion of Ukraine and the broader disruption to international relations caused by the war.

The paper by Christof Ruehl places a focus on key aspects of energy sanctions and the world economy — mandated versus unilateral sanctions. This analysis is in the context of the Russo-Ukrainian war and the series of sanctions imposed by Western economies, Japan, Australia, and others on Russia. The author points out that during the first 2 months of the conflict, the G7/NATO/EU/US have supported the efforts of Ukraine to fight the Russian invasion. In this context, a combined circa 30% of global GDP — the G7 share of world output — is squaring off against 11% of the world energy production, namely Russia’s share. An analytical focus is on the prospects of sanctioning Russia’s energy production and thereby to undermining Russia’s willingness to wage war against Ukraine. The author emphasizes that in the context of the Russo-Ukrainian war, sanctions differ from Western sanctions of the past: Sanctions and embargo proposals are now decentralized decisions made on a country-by-country basis and with “sanction picking” (i.e., no penalty for continuing to consume Russian energy). By contrast, previous sanctions on energy exports had been largely centralized and had a strong enforcement component, e.g., secondary sanctions attached to this. Which of these two approaches will be more successful in maximizing damage to Russian’s energy revenues while imposing minimal damage on the sanctioning countries? The author determines how a consistent strategy should look — an optimal path for energy sanctions should rely on decentralized, unilateral decision combined with other elements.

Finally, the paper by Werner Kirsch takes a closer look at the changes in the relative power balance within the European Union for the case of an enlargement to include Ukraine. The Banzhaf index is a very useful way to measure political power — in a quasi-game-theoretical context and considering potential coalition constellations amongst EU member countries — and the changes to be expected in the case of membership of the Ukraine in an enlarged EU28 are quite interesting to study: The main losers in terms of relative power changes — relevant with regard to qualified majority voting at the Council of the European Union — are the large EU countries; small EU countries stand to gain relative power in the context of an EU enlargement should there be the accession of Ukraine. The findings for an enlargement to include Ukraine raise new challenges for the European Union and could also bring about a new debate about adjusting the minimum majority requirement with respect to the population criterion (currently 65% of the EU27 population).

Combining selected papers from the EIIW’s 25th Anniversary workshop with recent papers on the Russo-Ukrainian war generates important and original economic insights and creates interesting analytical perspectives as well as new policy conclusions; for an understanding of the main implications for the new world economic order that might follow the end of the war, it is still too early, but economic, institutional, and mathematical/statistical analysis can already provide valuable insights into key issues surrounding the effects of this largely unexpected Russian invasion of Ukraine.

Clearly, the Russo-Ukrainian conflict raises not only analytical issues but also has many other perspectives, including the question of how strong individual Western countries should support the Ukraine in its defense vis-à-vis the aggressor. Germany, Italy, Spain, and other EU countries can be expected to raise defense expenditures relative to GDP and meet the 2% NATO target within a few years. Whether or not this will be decisive in forging a renewed solid transatlantic security cooperation remains to be seen.

One may hope for a quick round of diplomatic negotiations which could lead to a lasting peace — after which, the rebuilding of the Ukrainian economy would be only one of the major challenges faced in Europe. Certainly, individuals, governments, and International Organizations will be expected to support this process. While Ukraine and Poland had rather similar per capita incomes in 1991, the Polish economic recovery in the wake of the post-socialist recession was much faster than in Ukraine which, however, had a rather different privatization process (with a considerable role played by oligarchs) when compared to Poland. Governments in Warsaw put a considerable emphasis on pro-competition reforms in the privatization process.

The latter approach brought significant efficiency gains and EU membership — as a driver of institutional reforms even prior to membership in 2004 — also supported economic growth in Poland.

While post-war Ukraine, suffering from the shock of the war, might not be able to quickly reach the per capita income of Poland, it should have new opportunities for economic modernization and international integration once political stability and security can be restored. The benefits of adequate and appropriate reforms of Ukrainian institutions and a consistent economic policy could be rather significant; and it will be interesting to see whether or not the EU could come up with a type of Marshall Plan support program for Ukraine. Whether or not there will be an exodus of Western investors from Russia after the end of the war is not yet clear — the historical precedent of banks from Western European countries leaving Russia in the late nineteenth century may be considered to be a rather bad historical example here. Restoring the world economic order after the war will be a serious challenge and one may have to raise the question of how Western ideas and values can become a more attractive set of institutional elements in a modern Russia (and China) than they seemingly were over the three decades prior to the war. The fact that few politicians and research institutes in Europe seem to have drawn attention in a timely fashion to the impact that Russian philosopher Ivan Ilyin and other Russian thinkers from the nineteenth and early twentieth centuries have had on President Putin raises serious questions about the future research agenda on Russia and Europe, respectively (see, however, the book by the French philosopher Michel Eltchaninoff that already in 2015 has analyzed the background of Putin’s ideology — Eltchaninoff’s book was published in French in that year, a German edition followed in 2016 and an English edition in 2018).

A potential broad energy embargo of Western countries against Russia would transitorily reinforce the use of coal in OECD countries and thus reinforce the problem of global warming in a largely unanticipated manner. At the same time, a strong rise of fossil fuel prices in world markets will stimulate substitution dynamics in favor of renewable energy which naturally is an important topic for future research.

The EIIW gratefully acknowledges funding from the Schumpeter School Foundation at the University of Wuppertal towards the 25th Anniversary EIIW workshop. International Economics and economic policy issues will obviously rank highly on the agenda of industrialized — and developing countries — in the coming years; hopefully, scientists from all countries will be able to contribute to the analysis and the international debate.

